# Robust auto-weighted multi-view subspace clustering with common subspace representation matrix

**DOI:** 10.1371/journal.pone.0176769

**Published:** 2017-05-23

**Authors:** Wenzhang Zhuge, Chenping Hou, Yuanyuan Jiao, Jia Yue, Hong Tao, Dongyun Yi

**Affiliations:** 1 Department of Mathematics and System Science, National University of Defense Technology, Changsha, Hunan, China; 2 The College of Nine, National University of Defense Technology, Changsha, Hunan, China; 3 Key Laboratory, Taiyuan Satellite Launch Center, Taiyuan, Shanxi, China; Soochow University, CHINA

## Abstract

In many computer vision and machine learning applications, the data sets distribute on certain low-dimensional subspaces. Subspace clustering is a powerful technology to find the underlying subspaces and cluster data points correctly. However, traditional subspace clustering methods can only be applied on data from one source, and how to extend these methods and enable the extensions to combine information from various data sources has become a hot area of research. Previous multi-view subspace methods aim to learn multiple subspace representation matrices simultaneously and these learning task for different views are treated equally. After obtaining representation matrices, they stack up the learned representation matrices as the common underlying subspace structure. However, for many problems, the importance of sources and the importance of features in one source both can be varied, which makes the previous approaches ineffective. In this paper, we propose a novel method called Robust Auto-weighted Multi-view Subspace Clustering (RAMSC). In our method, the weight for both the sources and features can be learned automatically via utilizing a novel trick and introducing a sparse norm. More importantly, the objective of our method is a common representation matrix which directly reflects the common underlying subspace structure. A new efficient algorithm is derived to solve the formulated objective with rigorous theoretical proof on its convergency. Extensive experimental results on five benchmark multi-view datasets well demonstrate that the proposed method consistently outperforms the state-of-the-art methods.

## 1 Introduction

In many applications such as computer vision, data mining, pattern recognition and machine learning, there exists an assumption that the data points are drawn from multiple low-dimensional subspaces with each subspace corresponding to one category or class. Subspace clustering [1, 2] aims to explore the underlying subspace and cluster the data according to it.

Early subspace clustering methods can be roughly grouped into two categories: algebra based methods such as [3, 4], and statistics based methods such as [5, 6]. And recently, many methods [7–16] which belong to a new category, i.e., spectral clustering based [1] methods, have been proposed and these methods have achieved state-of-the-art performance. The core idea of spectral clustering based methods is to apply the self-representation property to compute affinities, i.e., represent every data point by a linear combination of other data points. However, these methods mostly focus on the features from single source rather than multiple ones.

In actual applications, data is often collected from diverse domains or obtained from different feature extractors, thus multi-view data are very common in many applications. For example, in computer vision, each image can be described by the color, texture, shapes and so on. In web mining, each web can be characterized by its content and link information, which are two distinct descriptions or views. In multi-lingual information retrieval, a document can be represented by several different languages. Since these different features can provide useful information from different views and these single-view subspace clustering methods have shown good performance, it is crucial to integrate these heterogeneous features to create more accurate robust multi-view subspace clustering methods.

More recently, a number of multi-view subspace clustering methods have been proposed [17–19]. The diversity-induced multi-view subspace clustering (DiMSC) was proposed in [17] to perform subspace clustering on different views simultaneously with a diverse term on the multiple representation matrices. The multi-view subspace clustering (MVSC) was introduced in [18] to perform clustering on the subspace representation of each view simultaneously with a common cluster structure. The low-rank tensor constrained multi-view subspace clustering (LT-MSC), which was proposed in [19], performs subspace clustering on different views simultaneously with a low rank tensor constraint, and the tensor is constructed by the subspace representation matrices. After obtaining subspace representation matrices, these methods use them to construct similarity matrices for different views independently and stack up these similarity matrices to a common one which represents the underlying common structures across different views. However, these methods neglect the different importance among views and the performance of their unified similarities may suffer when there is a less informative view.

In this paper, we try to solve the problem of subspace clustering for multi-view data. A novel method, named as Robust Auto-weighted Multi-view Subspace Clustering (RAMSC), has been presented. Different from the previous approaches [17–19] which treat different views equally and obtain a representation matrix in each view, our proposed method assigns a suitable weight for each view and purposes to learn a common representation matrix across different views to reflect the underlying common structure. Besides, the view weight factors can be tuned automatically and this process does not need any additional parameters. And by introducing an sparse norm, our proposed method is robust to the inaccurate features. We provide an effective algorithm to solve the proposed non-smooth minimization problem and prove that the algorithm will converge. In the algorithm, for each view, a feature weight matrix can be learned and we also proposes a new way to construct the common similarity matrix by utilizing the view weight factors and feature weight matrix. Compared to related state-of-the-art clustering methods, our proposed method consistently achieves better performance on five benchmark multi-view data sets.

The rest of this paper is organized as follows. Section 2 introduces the background and motivation of this paper. In Section 3, we propose our method RAMSC with a solving algorithm. In Section 4, we present some deep analyses about the proposed algorithm RAMSC, including convergence behavior, computational complexity and parameter determination. Experimental results and conclusions are shown in Section 5 and Section 6, respectively.

## 2 Background and motivation

In this section, first we introduce some notations, then briefly review the previous subspace clustering methods to show our research motivation.

### 2.1 Notations

Throughout this paper, vectors and matrices are written in boldface uppercase letters and boldface lowercase letters, respectively. For a vector **m**, the *ℓ*_2_-norm of vector **m** is denoted by ||**m**||_2_. And **m**^(*v*)^ denotes that **m** is derived from *v*-th view. For a matrix **M**, we denote its *i*-th row, *j*-th column and *ij*-th element as **m**_*i*:_, **m**_:*j*_ and *m*_*ij*_ respectively. The trace of matrix **M** is denoted by *Tr*(**M**). And we denote **M**^(*v*)^ as a matrix **M** derived from the *v*-th view representation. The *ℓ*_*r*,*p*_-norm of an matrix $M\in R^{d\times n}$ is defined as [20, 21]

$$\begin{array}{c} {\left||M|\right|}_{r,p}={\left(\sum_{i=1}^{d}{\left(\sum_{j=1}^{n}{\left|m_{ij}\right|}^{r}\right)}^{\frac{p}{r}}\right)}^{\frac{1}{p}},r>0,p>0. \end{array}$$ (1)

When *r* ≥ 1 and *p* ≥ 1, *ℓ*_*r*,*p*_-norm becomes a valid norm because it satisfies the three norm conditions.

### 2.2 Single-view and multi-view subspace clustering

Suppose $X=\left[x_{:1},x_{:2},...,x_{:n}\right]\in R^{d\times n}$ is the data matrix with *d*-dimensional features and *n* data points. The subspace clustering methods based on spectral clustering mainly have the following two steps:

First, the self-representation property [7] is used to represent data matrix **X** as

$$\begin{array}{c} X=\text{XZ}+E, \end{array}$$ (2)

where $Z=\left[z_{:1},z_{:2},...,z_{:n}\right]\in R^{n\times n}$ is the self-representation matrix with each **z**_:*i*_ being the representation of sample **x**_:*i*_, and **E** is the error matrix. The nonzero elements of **z**_:*i*_ correspond to points from the same subspace. And **Z** can be obtained by solving:

$$\begin{array}{c} \begin{array}{cc} \min_{Z}\; & ||X-{\text{XZ}||}_{l}+\lambda \Omega \left(Z\right),\\ s.t.\; & Z\in C \end{array} \end{array}$$ (3)

where || ⋅ ||_*l*_ can be considered a proper norm on error matrix **E**, Ω(**Z**) and C are the regularizer and constraint set on **Z**, respectively, and λ > 0 is a balance parameter. The existing methods [7–13] distinguish each other by employing different constraints or regularizers on **Z** or **E**;

Second, the obtained subspace structure **Z** is used to construct a similarity matrix **S** which encodes the pairwise similarity between data pairs by [22]

$$\begin{array}{c} S=\frac{|Z^{T}\left|+|Z\right|}{2}. \end{array}$$ (4)

Afterwards, spectral clustering algorithm [23] can be used on the computed similarity matrix **S** to get the final clustering results.

For multi-view data, suppose that *V* is the number of views and **X**^(1)^, **X**^(2)^, …, **X**^(*V*)^ are used to denote data matrix of each view, where $X^{\left(v\right)}\in R^{d^{\left(v\right)}\times n}$ for *v* = 1, 2, 3, …, *V* and *d*^(*v*)^ is the *v*-th view dimensionality. The single-view subspace clustering methods can not be applied on multi-view data to obtain a representation matrix directly. One naive strategy is to concatenate all the features together as a new view, and then employ single view methods on the concatenated features. However, this method ignores the difference among multiple views.

The previous multi-view subspace clustering methods consider that for each single view, a subspace representation $Z^{\left(v\right)}\in R^{n\times n}$ should be learned. They stack up these *V* tasks and focus on how to explore the relationships among these *V* representation matrices **Z**^(*v*)^ so that these **Z**^(*v*)^ can be learned simultaneously. Two more reasonable strategies in multi-view learning are adopted by them to achieve their goal: The first one is to explore complementary information from multiple views. DiMSC [17] explores the complementary of these representations **Z**^(*v*)^ by applying the Hilbert Schmidt independence criterion as a diversity term. LT-MSC [19] explores the complementary information from multiple views by regarding the subspace representation matrices **Z**^(*v*)^ as a tensor, then equipping the tensor with a low-rank constraint. The second strategy is to explore the consistence among multiple views. MVSC [18] explores the consistence of these representations **Z**^(*v*)^ by performing subspace clustering on individual modality respectively and then unifying them by a common indicator matrix.

After obtaining a representation **Z**^(*v*)^ for each view, all the above-mentioned multi-view subspace clustering methods construct a similarity matrix **S** by

$$\begin{array}{c} S=\sum_{v=1}^{V}\frac{|Z^{(v)T}\left|+\right|Z^{\left(v\right)}|}{2}. \end{array}$$ (5)

Then they apply spectral clustering algorithm [23] on **S** to obtain clustering results.

Although these multi-view subspace clustering methods have achieved good performance, there are mainly two drawbacks of these methods which leave room to improve the clustering performance:

1. These methods treat different views equally and neglect the different importance of different views. When they learn **Z**^(*v*)^, each view plays the same important role. When they construct the similarity matrix **S**, Eq (5) can be considered as $S=\sum_{v=1}^{V}S^{\left(v\right)}$, where **S**^(*v*)^ = (|**Z**^(*v*)*T*^| + |**Z**^(*v*)^|)/2 is a graph similarity matrix constructed from *v*-th view. This strategy may suffer when an unreliable similarity matrix is added to.
2. Eq (5) can also be considered as **S** = (|**Z**^*T*^| + |**Z**|)/2, where $\left|Z\right|=\sum_{v=1}^{V}\left|Z^{\left(v\right)}\right|$ can be considered as the underlying common structures across different views. The optimizing objectives of these methods are representation matrices **Z**^(*v*)^, however, the final clustering results are determined by the common structure **Z** which may brings such a drawback that these **Z**^(*v*)^ may have good properties because of the constraints or regularized terms, but **Z** may not keep these properties.

To address these two challenges, we will introduce our proposed novel multi-view subspace clustering method in next section.

## 3 Formulation and solution

In this section, we will first introduce the formulation of our method, and then an alternative algorithm will be presented to solve it.

### 3.1 Formulation

To overcome these two drawbacks, we propose a novel robust auto-weighted multi-view subspace clustering method. Our proposed method RAMSC utilizes a reasonable way to set view weight factors automatically and learns a common subspace representation **Z** which can be directly used to construct the common similarity matrix **S** across different views. Thus one important view can have a big weight, and the constraints or regularized terms can be set on **Z** which determines the final clustering results. The objective function of RAMSC is

$$\begin{array}{c} \min_{Z}\sum_{v=1}^{V}\sqrt{\left|\right|X^{\left(v\right)}-X^{\left(v\right)}Z{\left|\right|}_{2,p}^{p}+\lambda \Omega^{\left(v\right)}\left(Z\right)}, \end{array}$$ (6)

where λ is a tradeoff factor, $\left|\right|\cdot {\left|\right|}_{2,p}^{p}$ is the sparsity-inducing norm with 0 ≤ *p* ≤ 1 and each Ω^(*v*)^(**Z**) is a smooth regularized term. Denote the representation error matrix $E^{\left(v\right)}\in R^{d^{\left(v\right)}\times n}$ of the *v*-th view as

$$\begin{array}{c} E^{\left(v\right)}=X^{\left(v\right)}-X^{\left(v\right)}Z. \end{array}$$ (7)

The $\left|\right|\cdot {\left|\right|}_{2,p}^{p}$-norm of a matrix $E\in R^{d\times n}$ is defined as

$$\begin{array}{c} \left|\right|E^{\left(v\right)}{\left|\right|}_{2,p}^{p}=\sum_{i=1}^{d}{\left(\sum_{j=1}^{n}{\left|e_{ij}^{\left(v\right)}\right|}^{2}\right)}^{\frac{p}{2}}=\sum_{i=1}^{d}\left(|\right|e_{i:}^{\left(v\right)}{\left|\right|}_{2}{)}^{p}, \end{array}$$ (8)

where $e_{i:}^{\left(v\right)}$ is the *i*-th row of **E**^(*v*)^ and ||**E**^(*v*)^||_2,*p*_ is the *ℓ*_2,*p*_-norm as defined in Eq (1) with r = 2. Ω^(*v*)^(**Z**) aims to smooth the distribution of the common representation **Z** on the *v*-th view. These *v* smooth regularized terms Ω^(*v*)^(**Z**) enforce the common subspace representation matrix **Z** to meet the grouping effect. This analogous smooth regularized term is also employed by [13, 17, 24]. Specifically, in our method, each regularized term Ω^(*v*)^(**Z**) for *v* = 1, 2, …, *V* is defined as:

$$\begin{array}{c} \Omega^{\left(v\right)}\left(Z\right)=\frac{1}{2}\sum_{i=1}^{n}\sum_{j=1}^{n}w_{ij}^{\left(v\right)}\left|\right|z_{:i}-z_{:j}{\left|\right|}_{2}^{2}=Tr\left({\text{ZL}}^{\left(v\right)}Z^{T}\right), \end{array}$$ (9)

$W^{\left(v\right)}=\left(w_{ij}^{\left(v\right)}\right)$ is the weight matrix measuring the spatial closeness of the data points on *v*-th view. **L**^(*v*)^ = **D**^(*v*)^ − **W**^(*v*)^ is the Laplacian matrix, in which the degree **D**^(*v*)^ is the diagonal matrix with $d_{ii}^{\left(v\right)}=\sum_{j=1}^{n}w_{ij}^{\left(v\right)}$. **W**^(*v*)^ can be constructed by many different ways [25–29]. To show the robustness of our method, we construct 0-1 binary weighted *k*-nn graphs for each view and *k* is set to be 5 in all experiments.

Intuitively, there is no weight factor explicitly defined in Eq (6), and all different views are treated equally. By the following analysis, it can provide a reasonable way to learn the weight factors of each view. The Lagrange function of problem (6) can be written as

$$\begin{array}{c} \sum_{v=1}^{V}\sqrt{\left|\right|X^{\left(v\right)}-X^{\left(v\right)}Z{\left|\right|}_{2,p}^{p}+\lambda Tr\left({\text{ZL}}^{\left(v\right)}Z^{T}\right)}. \end{array}$$ (10)

Taking the derivative of Eq (10) with respect to **Z** and setting the derivative to zero, we have

$$\begin{array}{c} \sum_{v=1}^{V}\alpha^{\left(v\right)}\frac{\partial (||X^{\left(v\right)}-X^{\left(v\right)}Z|{|}_{2,p}^{p}+\lambda Tr\left({\text{ZL}}^{\left(v\right)}Z^{T}\right))}{\partial Z}=0, \end{array}$$ (11)

where

$$\begin{array}{c} \alpha^{\left(v\right)}=\frac{1}{2\sqrt{\left|\right|X^{\left(v\right)}-X^{\left(v\right)}Z{\left|\right|}_{2,p}^{p}+\lambda Tr\left({\text{ZL}}^{\left(v\right)}Z^{T}\right)}}. \end{array}$$ (12)

Eq (11) can not be directly solved because *α*^(*v*)^ is dependent on the target variable **Z**. However, if *α*^(*v*)^ is considered as the weight factor of the *v*-th view, and its value has been given or set to be stationary, Eq (11) can be considered as the solution of the following problem when these *α*^(*v*)^ are calculated or given:

$$\begin{array}{c} \min_{Z,\alpha^{\left(v\right)}}\sum_{v=1}^{V}\alpha^{\left(v\right)}\left(|\right|X^{\left(v\right)}-X^{\left(v\right)}{Z||}_{2,p}^{p}+\lambda Tr\left(ZL^{\left(v\right)}Z^{T}\right)). \end{array}$$ (13)

Solving the problem (13) to obtain the common representation matrix **Z** seems more reasonable. This problem can be considered as a sum of two parts with a tradeoff factor λ. The first part $\sum_{v=1}^{V}\alpha^{\left(v\right)}\left|\right|X^{\left(v\right)}-X^{\left(v\right)}Z{\left|\right|}_{2,p}^{p}$ is a linear combination of the subspace representation errors on each view. Increasing *α*^(*v*)^ tends to reduce representation error on the *v*-th view. The second part is to smooth **Z** on a linear combination of Laplacian matrices with suitable weights *α*^(*v*)^, i.e., $L=\sum_{v=1}^{V}\alpha^{\left(v\right)}L^{\left(v\right)}$. According to [30–32], the accuracy of **L** can be higher than that of each **L**^(*v*)^ or the sum of them $\sum_{v=1}^{V}L^{\left(v\right)}$.

Supposing that the common representation **Z** can be calculated from Eq (13), this **Z** can be used to update *α*^(*v*)^ according to Eq (12). Learning *α*^(*v*)^ in this way has following reasonable explanations and merits:

1. If *v*-th view is good, then $\left|\right|E^{\left(v\right)}{\left|\right|}_{2,p}^{p}$ and *Tr*(**Z** **L**^(*v*)^**Z**^*T*^) should be small, and thus according to Eq (12), the learned *α*^(*v*)^ is large.
2. The *ℓ*_2,*p*_-norm of **E**^(*v*)^ enforces the *ℓ*_*p*_-norm along the features direction of representation error matrix **E**^(*v*)^, and the *ℓ*_2_-norm along the data points direction. Thus, when 0 ≤ *p* ≤ 1, the effect of inaccurate features in the learning of *α*^(*v*)^ is reduced by the *ℓ*_*p*_-norm.
3. Unlike [31–35], which depends on an extra parameter to smooth the distribution of the view weights, learning *α*^(*v*)^ by Eq (12) has no parameter to handle and it naturally avoids the trivial solution.

Although the problem (13) has a more reasonable form to learn a good common **Z**, there are difficulties to solve it, which comes from the following two aspects: (1) the $\left|\right|\cdot {\left|\right|}_{2,p}^{p}$ terms are nonsmooth; (2) when *α*^(*v*)^ is calculated by Eq (12), *α*^(*v*)^ and **Z** are coupled with each other. In next subsection, we will propose an alternative algorithm to tackle them efficiently.

### 3.2 Optimization algorithm

To solve Eq (13), we consider the following problem to tackle the non-smooth norm problem:

$$\begin{array}{c} \min_{Z,U^{\left(v\right)},\alpha^{\left(v\right)}}\sum_{v=1}^{V}\alpha^{\left(v\right)}\left(H^{\left(v\right)}+\lambda Tr\left(ZL^{\left(v\right)}Z^{T}\right)\right), \end{array}$$ (14)

where

$$\begin{array}{c} H^{\left(v\right)}=Tr\left({\left(X^{\left(v\right)}-X^{\left(v\right)}Z\right)}^{T}U^{\left(v\right)}\left(X^{\left(v\right)}-X^{\left(v\right)}Z\right)\right). \end{array}$$ (15)

$U^{\left(v\right)}\in R^{d^{\left(v\right)}\times d^{\left(v\right)}}$ is the diagonal matrix corresponding to the *v*-th view and the *i*-th entry on the diagonal is defined as:

$$\begin{array}{c} u_{ii}^{\left(v\right)}=\frac{p}{2}\left|\right|e_{i:}^{\left(v\right)}{\left|\right|}_{2}^{p-2},\;\forall i=1,2,...,d^{\left(v\right)}, \end{array}$$ (16)

$u_{ii}^{\left(v\right)}$ is a subgradient of $\left|\right|E^{\left(v\right)}{\left|\right|}_{2,p}^{p}$ w.r.t. $e_{i:}^{\left(v\right)}$. To avoid the situation $e_{i:}^{\left(v\right)}=0$, which makes $u_{ii}^{\left(v\right)}=\frac{p}{2}{\left||\;0\;|\right|}_{2}^{p-2}$ can not be calculated when *p* < 2, in practice, we replace the *ℓ*_2,*p*_-norm with the regularize *ℓ*_2,*p*_-norm. And it is defined as:

$$\begin{array}{c} \left|\right|E^{\left(v\right)}{\left|\right|}_{2,p}={\left(\sum_{i=1}^{d}{\left({\left(e_{i:}^{\left(v\right)}\right)}^{T}\left(e_{i:}^{\left(v\right)}\right)+\epsilon \right)}^{\frac{p}{2}}\right)}^{\frac{1}{p}}, \end{array}$$ (17)

when *ϵ* → 0, the regularized $\left|\right|\cdot {\left|\right|}_{2,p}^{p}$ of **E**^(*v*)^ approximates the $\left|\right|E^{\left(v\right)}{\left|\right|}_{2,p}^{p}$. Thus $u_{ii}^{\left(v\right)}$ now can be regularized as

$$\begin{array}{c} u_{ii}^{\left(v\right)}=\frac{p}{2}{\left({\left(e_{i:}^{\left(v\right)}\right)}^{T}\left(e_{i:}^{\left(v\right)}\right)+\epsilon \right)}^{\frac{p-2}{2}}. \end{array}$$ (18)

This strategy avoids a bad situation, 0 on the denominator, and guarantees that we can repeat the following alternative steps.

- **The first step is fixing U**^(*v*)^ **and** *α*^(*v*)^, **updating the common subspace representation Z**.
  Differentiating the objective function *J* with respect to **Z** and setting it to zero
  $$\begin{array}{c} \text{AZ}+\text{ZB}+C=0, \end{array}$$ (19)
  where
  $$\begin{array}{c} \begin{array}{cc} & A=\sum_{v=1}^{V}\alpha^{\left(v\right)}X^{(v)T}U^{\left(v\right)}X^{\left(v\right)},\\ & B=\lambda \sum_{v=1}^{V}\alpha^{\left(v\right)}L^{\left(v\right)},\\ & C=-\sum_{v=1}^{V}\alpha^{\left(v\right)}X^{(v)T}U^{\left(v\right)}X^{\left(v\right)}. \end{array} \end{array}$$ (20)
  Eq (19) is a standard Sylvester equation, and according to [36], it has a unique optimal solution.
- **The second step is fixing** *α*^(*v*)^ **and Z, updating the feature weight matrix U**^(*v*)^ **for each view**.
  The representation error matrix **E**^(*v*)^ of each view is calculated by current **Z**, and then each diagonal element of **U**^(*v*)^ is updated by Eqs (16) or (18).
- **The third step is fixing Z and U**^(*v*)^**, updating the view weight factors** *α*^(*v*)^ **for each view** by Eq (12).

By the above three steps, we alternatively update **Z**, **U**^(*v*)^ as well as *α*^(*v*)^, and repeat the process iteratively. Until now, we can draw the following conclusions:

1. In the above procedures, **the alternating optimization converges**, and Z* which denotes the converged value of **Z** is at least **a local optimal solution to** Eq (6). (We will prove this conclusion in next section).
2. The second one is about initialization. Since these procedures can reach a local optimum of Eq (6), it is important to have a sensible initialization. We initialize all views with equal $\alpha^{\left(v\right)}=\frac{1}{V}$ as in previous approaches [37, 38]. And as in previous researches [21, 39], we initialize **U**^(*v*)^ = *I*^(*v*)^ since every feature on each view has the same importance at the beginning.

After obtaining the common self-representation matrix Z*, the similarity matrix **S**_1_ can be defined as

$$\begin{array}{c} S_{1}=\frac{|Z^{*}\left|+\right|Z^{*T}|}{2} \end{array}$$ (21)

and use the spectral clustering algorithm to produce the final clustering results, as has been adopted by traditional single-view subspace clustering methods.

Some single-view subspace clustering methods also use other ways to construct similarity matrix [13]. In this paper, to better exploit the merit of grouping effect, we further utilize the learned view weight factors *α*^(*v*)^ and feature weight matrices **U**^(*v*)^ to define a new similarity matrix **S**_2_ as

$$\begin{array}{c} S_{2}=\left({\left|\frac{z_{:i}^{*T}z_{:j}^{*}}{\left|\right|{\hat{x}}_{:i}{\left|\right|}_{2}\left|\right|{\hat{x}}_{:j}{\left|\right|}_{2}}\right|}^{\gamma }\right), \end{array}$$ (22)

where

$$\begin{array}{c} \left|\right|{\hat{x}}_{:i}{\left|\right|}_{2}=\sqrt{\sum_{v=1}^{V}\alpha^{\left(v\right)}\sum_{k=1}^{d^{\left(v\right)}}u_{kk}^{\left(v\right)}x_{k,i}^{(v)2}}. \end{array}$$ (23)

${\hat{x}}_{:i}={\left[\sqrt{\alpha^{\left(1\right)}}{\hat{x}}_{:i}^{(1)T},...,\sqrt{\alpha^{V}}{\hat{x}}_{:i}^{(V)T}\right]}^{T}\in R^{\sum_{v}^{V}d^{\left(v\right)}}$ denotes the new *i*-th data point which concatenates re-weighted features, and ${\hat{x}}_{:i}^{\left(v\right)}={\left[\sqrt{u_{11}^{\left(v\right)}}x_{1i}^{\left(v\right)},...,\sqrt{u_{d^{\left(v\right)}d^{\left(v\right)}}^{\left(v\right)}}x_{d^{\left(v\right)}i}^{\left(v\right)}\right]}^{T}\in R^{d^{\left(v\right)}}$ is the re-weighted feature on the *v*-th view. *γ* > 0 is utilized to control the similarity variances. The new similarity measure can be considered as the inner product of the new common representation vectors normalized by the norms of their new features which are weighted by view weight factors *α*^(*v*)^ and feature weight matrices **U**^(*v*)^.

Based on the above analysis, we summarize the procedures of our method RAMSC in Algorithm 1.

**Algorithm 1** Algorithm to solve RAMSC in Eq (6)

**Input:**

1. Data for *V* views {**X**^(1)^, ⋯,**X**^(*V*)^} and $X^{\left(v\right)}\in R^{d^{\left(v\right)}\times n}$

2. The expected number of clusters *c*,

3. The parameter λ, *p*, *k* and *γ*.

**Initialize:**

1. Initialize the feature weight matrix **U**^(*v*)^ = *I*^(*v*)^ for each view, where $I^{\left(v\right)}\in R^{d^{\left(v\right)}\times d^{\left(v\right)}}$ is the identity matrix.

2. Initialize the view weight factor $\alpha^{\left(v\right)}=\frac{1}{V}$ for each view.

3. Build the 0-1 weighted *k*-nn graphs **W**^(*v*)^ and compute the corresponding Laplacian matrices **L**^(*v*)^ for each view.

**while** not converged **do**

 1. Compute the common representations **Z** by solving the Sylvester eq (19).

 2. Update the diagonal feature weight matrix **U**^(*v*)^ for each view. Its diagonal elements can be updated by Eqs (16) or (18).

 3. Update the view weight factor *α*^(*v*)^ for each view by Eq (12).

**end while**

4. Compute similarity matrix by either Eqs (21) or (22).

5. Use spectral clustering algorithm to obtain *c* clusters.

**Output:** Clustering result.

## 4 Performance analysis

### 4.1 Convergence analysis

To prove that the proposed Algorithm 1 converges and it can reach at least a local optimal solution of Eq (6), we first need to introduce the following lemma [21].

**Lemma 1** *When* 0 < *p* ≤ 2, *for any positive number a and b, the inequality holds:*

$$\begin{array}{c} a^{p}-\frac{p}{2}\frac{a^{2}}{b^{2-p}}\;⩽\;b^{p}-\frac{p}{2}\frac{b^{2}}{b^{2-p}}. \end{array}$$ (24)

**Theorem 1** *Each updated* **Z** *in Alg. 1 will monotonically decrease the objective of the* problem (13) *in each iteration*.

**Proof:** Denote $\tilde{Z}$ as the updated **Z** in each iteration and ${\tilde{E}}^{\left(v\right)}=X^{\left(v\right)}-X^{\left(v\right)}\tilde{Z}$ is the *v*-th representation error matrix calculated by $\tilde{Z}$. According to the optimization to $\tilde{Z}$ in Alg. 1, $\tilde{Z}$ reaches the unique optimal solution of the problem (14) when *α*^(*v*)^ and **U**^(*v*)^ are fixed, so

$$\begin{array}{c} \begin{array}{cc} & \sum_{v=1}^{V}\alpha^{\left(v\right)}\left(Tr\left({\tilde{E}}^{(v)T}U^{\left(v\right)}{\tilde{E}}^{\left(v\right)}\right)+\lambda Tr\left(\tilde{Z}L^{\left(v\right)}{\tilde{Z}}^{T}\right)\right)\\ ⩽\; & \sum_{v=1}^{V}\alpha^{\left(v\right)}\left(Tr\left(E^{(v)T}U^{\left(v\right)}E^{\left(v\right)}\right)+\lambda Tr\left(ZL^{\left(v\right)}Z^{T}\right)\right). \end{array} \end{array}$$ (25)

Combining weight matrix **U**^(*v*)^ which $u_{ii}^{\left(v\right)}=\frac{p}{2}\left|\right|e_{i:}^{\left(v\right)}{\left|\right|}_{2}^{p-2}$, this inequation can be rewritten as:

$$\begin{array}{c} \begin{array}{cc} & \sum_{v=1}^{V}\alpha^{\left(v\right)}\left(\sum_{i=1}^{d^{\left(v\right)}}\frac{p}{2}\frac{\left|\right|{\tilde{e}}_{i:}^{\left(v\right)}{\left|\right|}_{2}^{2}}{\left|\right|e_{i:}^{\left(v\right)}{\left|\right|}_{2}^{2-p}}+\lambda Tr\left(\tilde{Z}L^{\left(v\right)}{\tilde{Z}}^{T}\right)\right)\\ ⩽\; & \sum_{v=1}^{V}\alpha^{\left(v\right)}\left(\sum_{i=1}^{d^{\left(v\right)}}\frac{p}{2}\frac{\left|\right|e_{i:}^{\left(v\right)}{\left|\right|}_{2}^{2}}{\left|\right|e_{i:}^{\left(v\right)}{\left|\right|}_{2}^{2-p}}+\lambda Tr\left(ZL^{\left(v\right)}Z^{T}\right)\right). \end{array} \end{array}$$ (26)

Generally, $\left|\right|e_{i:}^{\left(v\right)}{\left|\right|}_{2}>0$ and $\left|\right|{\tilde{e}}_{i:}^{\left(v\right)}{\left|\right|}_{2}>0$, and the regularized *ℓ*_2,*p*_-norm can be used to guarantee it. According to Lemma 1, we can derive

$$\begin{array}{c} \left|\right|{\tilde{e}}_{i:}^{\left(v\right)}{\left|\right|}_{2}^{p}-\frac{p}{2}\frac{\left|\right|{\tilde{e}}_{i:}^{\left(v\right)}{\left|\right|}_{2}^{2}}{\left|\right|e_{i:}^{\left(v\right)}{\left|\right|}_{2}^{2-p}}\;⩽\;\left|\right|e_{i:}^{\left(v\right)}{\left|\right|}_{2}^{p}-\frac{p}{2}\frac{\left|\right|e_{i:}^{\left(v\right)}{\left|\right|}_{2}^{2}}{\left|\right|e_{i:}^{\left(v\right)}{\left|\right|}_{2}^{2-p}}. \end{array}$$ (27)

Thus the following inequality holds

$$\begin{array}{c} \begin{array}{cc} & \sum_{v=1}^{V}\alpha^{\left(v\right)}\sum_{i=1}^{d^{\left(v\right)}}\left|\right|{\tilde{e}}_{i:}^{\left(v\right)}{\left|\right|}_{2}^{p}-\sum_{v=1}^{V}\alpha^{\left(v\right)}\sum_{i=1}^{d^{\left(v\right)}}\frac{p}{2}\frac{\left|\right|{\tilde{e}}_{i:}^{\left(v\right)}{\left|\right|}_{2}^{2}}{\left|\right|e_{i:}^{\left(v\right)}{\left|\right|}_{2}^{2-p}}\\ ⩽\; & \sum_{v=1}^{V}\alpha^{\left(v\right)}\sum_{i=1}^{d^{\left(v\right)}}\left|\right|e_{i:}^{\left(v\right)}{\left|\right|}_{2}^{p}-\sum_{v=1}^{V}\alpha^{\left(v\right)}\sum_{i=1}^{d^{\left(v\right)}}\frac{p}{2}\frac{\left|\right|e_{i:}^{\left(v\right)}{\left|\right|}_{2}^{2}}{\left|\right|e_{i:}^{\left(v\right)}{\left|\right|}_{2}^{2-p}}. \end{array} \end{array}$$ (28)

Summing Eqs (26) and (28), we have

$$\begin{array}{c} \begin{array}{c} \sum_{v=1}^{V}\alpha^{\left(v\right)}\left(|\right|X^{\left(v\right)}-X^{\left(v\right)}\tilde{Z}{\left|\right|}_{2,p}^{p}+\lambda Tr\left(\tilde{Z}L^{\left(v\right)}{\tilde{Z}}^{T}\right))\\ ⩽\;\sum_{v=1}^{V}\alpha^{\left(v\right)}\left(|\right|X^{\left(v\right)}-X^{\left(v\right)}{Z||}_{2,p}^{p}+\lambda Tr\left(ZL^{\left(v\right)}Z^{T}\right)). \end{array} \end{array}$$ (29)

Thus the objective of the problem (13) has been decreased by $\tilde{Z}$ in each iteration.

**Theorem 2** *Each updated* **Z** *in Alg. 1 will monotonically decrease the objective of the* problem (6) *in each iteration, which makes converged* Z* *be a local optimal solution*.

**Proof:** Denote $L^{\left(v\right)}\left(Z\right)$ as

$$\begin{array}{c} L^{\left(v\right)}\left(Z\right)=\sqrt{\left|\right|X^{\left(v\right)}-X^{\left(v\right)}Z{\left|\right|}_{2,p}^{p}+\lambda Tr\left(ZL^{\left(v\right)}Z^{T}\right)}. \end{array}$$ (30)

According to Eq (29), $\tilde{Z}$ makes the objective of Eq (13) have smaller value than **Z**. Combining view weight factors $\alpha^{\left(v\right)}=1/2L^{\left(v\right)}\left(Z\right)$, we can derive

$$\begin{array}{c} \begin{array}{c} \sum_{v=1}^{V}\frac{1}{2}\frac{{\left(L^{\left(v\right)}\left(\tilde{Z}\right)\right)}^{2}}{L^{\left(v\right)}\left(Z\right)}⩽\;\sum_{v=1}^{V}\frac{1}{2}\frac{{\left(L^{\left(v\right)}\left(Z\right)\right)}^{2}}{L^{\left(v\right)}\left(Z\right)}. \end{array} \end{array}$$ (31)

Since $L^{\left(v\right)}\left(\tilde{Z}\right)>0$ and $L^{\left(v\right)}\left(Z\right)>0$, according to Lemma 1, when *p* = 1, we have

$$\begin{array}{c} \begin{array}{c} \sum_{v=1}^{V}L^{\left(v\right)}\left(\tilde{Z}\right)-\sum_{v=1}^{V}\frac{1}{2}\frac{{\left(L^{\left(v\right)}\left(\tilde{Z}\right)\right)}^{2}}{L^{\left(v\right)}\left(Z\right)}⩽\;\sum_{v=1}^{V}L^{\left(v\right)}\left(Z\right)-\sum_{v=1}^{V}\frac{1}{2}\frac{{\left(L^{\left(v\right)}\left(Z\right)\right)}^{2}}{L^{\left(v\right)}\left(Z\right)}. \end{array} \end{array}$$ (32)

Summing Eqs (31) and (32), we arrive at

$$\begin{array}{c} \begin{array}{cc} & \sum_{v=1}^{V}\sqrt{\left|\right|X^{\left(v\right)}-X^{\left(v\right)}\tilde{Z}{\left|\right|}_{2,p}^{p}+\lambda Tr\left(\tilde{Z}L^{\left(v\right)}{\tilde{Z}}^{T}\right)}\\ ⩽\; & \sum_{v=1}^{V}\sqrt{\left|\right|X^{\left(v\right)}-X^{\left(v\right)}Z{\left|\right|}_{2,p}^{p}+\lambda Tr\left(ZL^{\left(v\right)}Z^{T}\right)}. \end{array} \end{array}$$ (33)

Thus the alternating optimization will monotonically decrease the objective of the problem (6) in each iteration until it converges. In the convergence, the converged Z* satisfy the Eq (11) which is the KKT condition of problem (6). Therefore, Z* is at least a local optimal solution of the problem (6).

### 4.2 Computational complexity and parameter determination

As seen from the procedure of RAMSC in Algorithm 1, we have solved this problem in an alternative way. The computational complexity in solving each problem is listed as follows. (1) The problem in Eq (19) can be solved by the Bartels-Stewart algorithm which has a computational complexity of $O(n^{3})$; (2) The problem in Eq (16) can be effectively solved by computing the 2-norm of a vector. The computational complexity is $O(\sum_{v=1}^{V}{\left(d^{\left(v\right)}\right)}^{2})$; (3) Solving the problem in Eq (12) to update the optimal weight for each view has complexity $O(n^{2}\times V)$. In summary, the total computational complexity of RAMSC is $O(T\times \max\left\{n^{3},\sum_{v=1}^{V}{\left(d^{\left(v\right)}\right)}^{2}\right\})$, where *T* is the number of iteration.

Since parameter determination is still an open problem [40, 41], we determine the parameters of our method empirically as in previous researches. As for *p*, it is designed to add sparsity to representation error matrices **E**^(*v*)^ which can alleviate the effect of inaccurate features in the learning of *α*^(*v*)^. Paper [43] is a timely and comprehensive survey, and a very good material to master the sparse learning field. According to it, we set *p* = 1, and this setting has been proven to be effective in most applications [20, 42]. As for *k*, it is the neighbor number to construct graphs **W**^(*v*)^. Methods [13, 17] using similar regularized terms perform stably with different *k*, so we construct 5-nn graphs.

As for the parameter λ, it is very vital to the final performance since it is employed to balance the self representation accuracy and the smoothness of **Z**. Since there is no prior information about λ, we determine it by grid search in a heuristic way as in previous researches [13, 17, 42]. Concretely, λ is tuned from 1,2 and 5 to 60 with an incremental step 5 to get the best λ. When Eq (22) is used to construct the similarity matrix, we search it from 0.1 to 2 with an incremental step 0.2 to get the best *γ*.

## 5 Experiments

In this section, our proposed RAMSC has been evaluated on five widely used data sets, and some numerical results of its convergency behaviors and also have been shown.

### 5.1 Data set descriptions

To validate the effectiveness of our method, we use five multiview benchmark datasets. They are various kinds of data arisen in many real applications with different characters and commonly used in multiple view learning. They are Microsoft Research Cambridge Volume 1 (MSRC-v1) [44], Caltech101 [45], NBA-NASCAR [46], Handwritten Dutch Digit Recognition (Digit) [47] and Web Knowledge Base (WebKB) [48]. The statistics information of the five data sets is concluded in Table 1 and the detailed information about them is shown as the following

- **MSRC-v1** data set consists 240 images and is divided into 8 classes. Following [49], we select 7 classes composed of tree, building, airplane, cow, face, car, bicycle and each class has 30 images. To distinguish all of scenes, we extract 256 Local Binary Pattern (LBP), 100 Histogram of Oriented Gradient (HOG), 512 GIST, 1302 CENTRIST, 48 Color Moment (CMT) and 200 SIFT features.
- **Caltech101-7** data set is composed of 8677 objective images which belong to 101 categories. We selected 7 widely used classes, including DollaBill, Faces, Garfield, Motorbikes, Snoopy, Stop-Sign and Windsor-Chair. Following [50], the data set has totally 441 images. In order to obtain different views, we extract 256 LBP, 100 PyramidHOG (PHOG), 512 GIST, 32 Gabor texture, 200 SURF and 200 SIFT features.
- **Digit** data set contains 2,000 data points for 0 to 9 ten digit classes and each class has 200 data points. Six published features can be used for multi-view clustering: 76 Fourier coefficients of the character shapes (FOU), 216 profile correlations (FAC), 64 Karhunen-love coefficients (KAR), 240 pixel averages in 2 × 3 windows (PIX), 47 Zernike moment (ZER) and 6 morphological (MOR) features.
- **NBA-NASCAR** data set is collected from the sports gallery of the yahoo! website in 2008. Following [46], this data set consists 420 NBA images and 420 NASCAR images. For each image, there an attached short text describing information. To get different views, each image is normalized to have 1024 gray features, and from each text, 296 TFIDF features have been extracted.
- **WebKB** data set is a subset of web documents from four universities. This data set consists 1051 pages which are classified 2 classes: 230 Course pages and 821 Non-Course pages. Each page has 2 views: Fulltext view contains 2949 features representing the textual content on the web page, and Inlinks view consists 334 features recording that the anchor text on the hyperlinks pointing to the pages.

**Table 1:** Details of the multiview datasets used in our experiments (view type (dimensionality)).

View type	MSRC-v1	Caltech101-7	Digit	NBA-NASCAR	WebKB
1	LBP (256)	LBP (256)	FOU (76)	Gray (1024)	Fulltext (2949)
2	HOG (100)	PHOG (680)	FAC (216)	TFIDF (296)	Inlinks (334)
3	GIST (512)	GIST (512)	KAR (64)	-	-
4	CENTRIST (1302)	Gabor (32)	PIX (240)	-	-
5	CMT (48)	SURF (200)	ZER (47)	-	-
6	SIFT (200)	SIFT (200)	MOR (6)	-	-
Date points	210	441	2000	840	1051
Classes	7	7	10	2	2

### 5.2 Experimental setup

To evaluate the performance of our method, we have compared our method with each single view counterpart. Single view methods on the concatenated features are also compared. Besides, we compare with other state-of-the-art methods, including robust multi-view K-means clustering (RMKMC) [33], pair-wised co-regularized multi-modal spectral clustering (PC-SPC) [30], centroid co-regularized multi-modal spectral clustering (CC-SPC) [30], multi-view subspace clustering (MVSC) [18] and diversity induced multi-view subspace clustering (DiMSC) [17].

- **SPC:** We employ the standard spectral clustering (SPC) [23] algorithm directly on each view, and report the results as baselines.
- **SMR:** We first run smooth representation clustering (SMR) [13] on each view features to get the subspace representations, and then run spectral clustering on such representations.
- **SPC-CON** and **SMR-CON:** We first concatenate all features together as a new single view, and then run SPC [23] and SMR [13] respectively on it.
- **RMKMC:** The robust multi-view K-means clustering method obtains the common cluster indicators across multiple views by minimizing the linear combination of the relaxed K-means on each view with learned weight factors.
- **PC-SPC:** This method enforces the corresponding point in different modality to have the same cluster membership by a pair-wised co-regularization term, which makes different views be same to each other.
- **CC-SPC:** This method is similar to PC-SPC, other than a centroid-based co-regularization term, which makes different views be same to a common one.
- **MVSC:** This method perform subspace clustering on individual modality respectively and then unify them with a common indicator matrix.
- **DiMSC:** This method learns subspace representations and employs the Hilbert-Schmidt Independence Criterion to enhance complementary information.

For fair comparison, we download the source codes of the compared methods from the authors’ websites and follow their experimental settings and the parameter tuning steps in their papers to get their best parameters. And for RAMSC, we construct 0-1 binary 5-nn graphs **W**^(*v*)^ for each view and the *p* is fixed 1 in all experiments. Thus only one parameter λ in our method needs to be tuned. We search the best λ from 1, 2 and 5 to 60 with incremental step 5. RAMSC(*S*_2_) denotes that we use Eq (22) to construct **S**_2_, and the best parameter *γ* is searched from 0.1 to 2 with incremental step 0.2. And the experimental results are corresponding to their best parameters.

Before we do the clustering work, we first normalize each view of the multi-view data to make all the values in the range [−1, 1]. All the experiments are repeated 50 times independently, and the mean and standard deviation of the results are reported.

Three standard clustering evaluation metrics are utilized to measure the multi-view clustering performance, that is, **Clustering Accuracy (ACC)**, **Normalized Mutual Information (NMI)** and **Purity**.

### 5.3 Experimental results

The experiment results of the five datasets with three metrics are shown in Tables 2, 3, 4, 5 and 6. In terms of the clustering accuracy, we have the following observations.

1. From Tables 2, 3, 4, 5 and 6, we conclude that our proposed method outperforms the competing methods on all the benchmark datasets. And although ACC, NMI and Purity are three different evaluation metrics, they all indicate the advantages of our method. The clustering results show the effectiveness of the way to construct similarity matrix by Eq (22), and compared the way of Eq (21), it can achieve better or at least comparable performance.
2. From Tables 2, 3 and 4, it can be seen that some individual view features are more discriminative for performing clustering. And as for the comparison between single view methods and previous multi-view approaches, the previous multi-view clustering methods can not always achieve better performances. This may be caused by the fact that previous methods characterize the structures of each view data separately and combine them by simply addition operations, which makes the final clustering results affect by these inaccurate structures. Our approach can perform better than single view methods in most cases because our method distributes small weight factors for inaccurate views and learns a common self representation matrix **Z** which can be used to construct a common similarity matrix **S** among different views.
3. Tables 5 and 6 show the robustness of our method. On NBA-NASCAR data set, all the competing methods except RMKMC can not achieve reasonable performance. It is because that RMKMC utilizes a weight factor for each view and the sparsity-inducing norm to eliminate the influence of the outliers, while the other competing methods do not consider the ouliers and sparsity of the input data. Compared with RMKMC, our method learns view weight factors automatically without an additional parameter and use the $\left|\right|\cdot {\left|\right|}_{2,p}^{p}$ norm to eliminate the influence of the inaccurate features. Our method has better performance on NBA-NASCAR data set and still achieve good performance on WebKB data set when all other compared methods do not work.

**Table 2:** Clustering results of different methods on MSRC-v1 data set. (mean(± std)). (On the following five result tables, two best results of each metrics are bold).

Method	ACC	NMI	Purity
SC(1)	0.6022(±0.0510)	0.4887(±0.0283)	0.6336(±0.0382)
SC(2)	0.5755(±0.0230)	0.4936(±0.0262)	0.5965(±0.0245)
SC(3)	0.6547(±0.0369)	0.5865(±0.0284)	0.6877(±0.0377)
SC(4)	0.7002(±0.0567)	0.6064(±0.0330)	0.7096(±0.0430)
SC(5)	0.2873(±0.0124)	0.1448(±0.0146)	0.3174(±0.0122)
SC(6)	0.5489(±0.0371)	0.4538(±0.0311)	0.5750(±0.0280)
SMR(1)	0.6166(±0.0351)	0.4853(±0.0307)	0.6388(±0.0356)
SMR(2)	0.6184(±0.0347)	0.4958(±0.0242)	0.6283(±0.0296)
SMR(3)	0.7074(±0.0352)	0.6444(±0.0248)	0.7490(±0.0246)
SMR(4)	0.7596(±0.0742)	0.7106(±0.0373)	0.7733(±0.0556)
SMR(5)	0.4932(±0.0384)	0.3765(±0.0291)	0.5101(±0.0330)
SMR(6)	0.5538(±0.0279)	0.4605(±0.0259)	0.5752(±0.0264)
SC-CON	0.5983(±0.0337)	0.4796(±0.0223)	0.6192(±0.0287)
SMR-CON	0.7338(±0.0495)	0.6920(±0.0232)	0.7661(±0.0326)
RMKMC	0.6501(±0.0782)	0.5700(±0.0537)	0.6728(±0.0651)
PC-SPC	0.7936(±0.0589)	0.6965(±0.0278)	0.8029(±0.0433)
CC-SPC	0.8368(±0.0605)	0.7799(±0.0306)	0.8546(±0.0393)
MVSC	0.7444(±0.0754)	0.7076(±0.0507)	0.7626(±0.0608)
DiMSC	0.7759(±0.0462)	0.6788(±0.0361)	0.7825(±0.0382)
RAMSC	**0.9078**(±0.0534)	**0.8462**(±0.0326)	**0.9125**(±0.0401)
RAMSC(*S*_2_)	**0.9149**(±0.0480)	**0.8512**(±0.0260)	**0.9184**(±0.0359)

**Table 3:** Clustering results of different methods on Caltech101-7 data set. (mean(± std)).

Method	ACC	NMI	Purity
SC(1)	0.4208(±0.0228)	0.3188(±0.0253)	0.5233(±0.0242)
SC(2)	0.4699(±0.0288)	0.3839(±0.0385)	0.5653(±0.0299)
SC(3)	0.6116(±0.0515)	0.5248(±0.0490)	0.6644(±0.0441)
SC(4)	0.5275(±0.0342)	0.4348(±0.0379)	0.5746(±0.0276)
SC(5)	0.6265(±0.0371)	0.5787(±0.0211)	0.7031(±0.0274)
SC(6)	0.5208(±0.0225)	0.4453(±0.0271)	0.5965(±0.0218)
SMR(1)	0.3288(±0.0197)	0.1602(±0.0127)	0.3936(±0.0097)
SMR(2)	0.4946(±0.0303)	0.4645(±0.0261)	0.6009(±0.0303)
SMR(3)	0.6874(±0.0336)	0.6331(±0.0271)	0.7639(±0.0336)
SMR(4)	0.5498(±0.0218)	0.4558(±0.0244)	0.5837(±0.0221)
SMR(5)	0.5212(±0.0587)	0.4631(±0.0355)	0.6095(±0.0357)
SMR(6)	0.5191(±0.0229)	0.4090(±0.0279)	0.6089(±0.0274)
SC-CON	0.4303(±0.0258)	0.3230(±0.0284)	0.5317(±0.0246)
SMR-CON	0.6247(±0.0246)	0.6049(±0.0288)	0.7095(±0.0343)
RMKMC	0.6034(±0.0680)	0.5488(±0.0482)	0.6846(±0.0541)
PC-SPC	0.6975(±0.0499)	0.6547(±0.0262)	0.7581(±0.0288)
CC-SPC	0.7047(±0.0654)	0.6879(±0.0378)	0.7972(±0.0389)
MVSC	0.6034(±0.0309)	0.4766(±0.0373)	0.6559(±0.0314)
DiMSC	0.7312(±0.0244)	0.6458(±0.0179)	0.7698(±0.0268)
RAMSC	**0.7384**(±0.0082)	**0.7276**(±0.0080)	**0.8258**(±0.0115)
RAMSC(*S*_2_)	**0.7512**(±0.0171)	**0.7388**(±0.0110)	**0.8381**(±0.0166)

**Table 4:** Clustering results on dight data set. (mean(± std)).

Method	ACC	NMI	Purity
SC(1)	0.5714(±0.0484)	0.6061(±0.0256)	0.6307(±0.0347)
SC(2)	0.8329(±0.0683)	0.8181(±0.0337)	0.8493(±0.0549)
SC(3)	0.6651(±0.0438)	0.7804(±0.0249)	0.7457(±0.0355)
SC(4)	0.8144(±0.0705)	0.8374(±0.0299)	0.8476(±0.0506)
SC(5)	0.5758(±0.0389)	0.6273(±0.0217)	0.6139(±0.0356)
SC(6)	0.5978(±0.0389)	0.6159(±0.0217)	0.6179(±0.0356)
SMR(1)	0.6210(±0.0234)	0.5937(±0.0155)	0.6374(±0.0167)
SMR(2)	0.8555(±0.0632)	0.8119(±0.0308)	0.8666(±0.0504)
SMR(3)	0.7890(±0.0576)	0.7625(±0.0272)	0.8007(±0.0476)
SMR(4)	0.8697(±0.0722)	0.8375(±0.0302)	0.8852(±0.0522)
SMR(5)	0.3505(±0.0116)	0.2850(±0.0092)	0.3573(±0.0071)
SMR(6)	0.4628(±0.0145)	0.4533(±0.0141)	0.4889(±0.0138)
SC-CON	0.8729(±0.0698)	0.8756(±0.0326)	0.8847(±0.0598)
SMR-CON	0.8496(±0.0725)	0.8344(±0.0288)	0.8720(±0.0501)
RMKMC	0.7853(±0.0800)	0.8125(±0.0384)	0.8190(±0.0614)
PC-SPC	0.8682(±0.0604)	0.8267(±0.0303)	0.8759(±0.0500)
CC-SPC	0.8768(±0.0605)	0.8234(±0.0338)	0.8855(±0.0471)
MVSC	0.8242(±0.0686)	0.8399(±0.0355)	0.8286(±0.0664)
DiMSC	0.8400(±0.0569)	0.8076(±0.0347)	0.8465(±0.0518)
RAMSC	**0.9299**(±0.0439)	**0.8864**(±0.0199)	**0.9343**(±0.0333)
RAMSC(*S*_2_)	**0.9173**(±0.0611)	**0.8886**(±0.0310)	**0.9229**(±0.0518)

**Table 5:** Clustering results on NBA-NASCAR data set. (mean(± std)).

Method	ACC	NMI	Purity
SC(1)	0.5631(±0.0000)	0.0139(±0.0000)	0.5631(±0.0000)
SC(2)	0.5036(±0.0000)	0.0003(±0.0000)	0.5036(±0.0000)
SMR(1)	0.6440(±0.0000)	0.0640(±0.0000)	0.6440(±0.0000)
SMR(2)	0.5750(±0.0000)	0.0289(±0.0000)	0.5750(±0.0000)
SC-CON	0.5631(±0.0000)	0.0132(±0.0000)	0.5631(±0.0000)
SMR-CON	0.7952(±0.0000)	0.2712(±0.0000)	0.7952(±0.0000)
RMKMC	0.9858(±0.0000)	0.9005(±0.0000)	0.9858(±0.0000)
PC-SPC	0.7250(±0.0000)	0.1521(±0.0000)	0.7250(±0.0000)
CC-SPC	0.8357(±0.0000)	0.3555(±0.0000)	0.8357(±0.0000)
MVSC	0.5131(±0.0000)	0.0060(±0.0000)	0.5131(±0.0000)
DiMSC	0.5476(±0.0000)	0.0071(±0.0000)	0.5476(±0.0000)
RAMSC	**0.9893**(±0.0000)	**0.9146**(±0.0000)	**0.9893**(±0.0000)
RAMSC(*S*_2_)	**0.9917**(±0.0000)	**0.9316**(±0.0000)	**0.9917**(±0.0000)

**Table 6:** Clustering results on WebKB data set. (mean(± std)).

Method	ACC	NMI	Purity
SC(1)	0.7774(±0.0000)	0.0013(±0.0000)	0.7812(±0.0000)
SC(2)	0.7755(±0.0000)	0.0027(±0.0000)	0.7812(±0.0000)
SMR(1)	0.7402(±0.0000)	0.0573(±0.0000)	0.7812(±0.0000)
SMR(2)	0.8069(±0.0000)	0.0665(±0.0000)	0.8069(±0.0000)
SC-CON	0.7774(±0.0000)	0.0013(±0.0000)	0.7812(±0.0000)
SMR-CON	0.7774(±0.0000)	0.0013(±0.0000)	0.7812(±0.0000)
RMKMC	0.8049(±0.0000)	0.1592(±0.0000)	0.8159(±0.0000)
PC-SPC	0.7659(±0.0000)	0.0991(±0.0000)	0.7812(±0.0000)
CC-SPC	0.5785(±0.0000)	0.0019(±0.0000)	0.7812(±0.0000)
MVSC	0.7802(±0.0000)	0.0041(±0.0000)	0.7812(±0.0000)
DiMSC	0.6147(±0.0000)	0.0006(±0.0000)	0.7812(±0.0000)
RAMSC	**0.9401**(±0.0000)	**0.5689**(±0.0000)	**0.9401**(±0.0000)
RAMSC(*S*_2_)	**0.9439**(±0.0000)	**0.5889**(±0.0000)	**0.9439**(±0.0000)

### 5.4 Convergence behavior

In order to verify the convergence of Algorithm 1, we present the numerical results of the convergence behavior on datasets MSRC-v1 and Caltech101-7.

**Fig 1:**
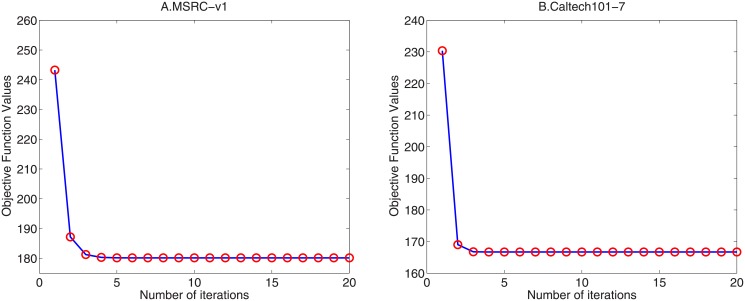
Convergence behaviors of RAMSC with λ = 50 on two datasets. (A) MSRC-v1; (B) Caltech101-7.

The convergence curves are displayed in Fig 1. As shown in Fig 1, the objective values of Eq (6) are non-increasing during the iterations and converge to a fixed value. Additionally, our algorithm converges within 10 iterations which means it has fast convergence speed.

### 5.5 Parameter determination

**Fig 2:**
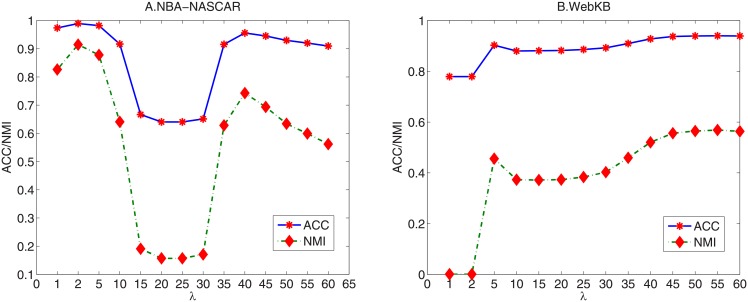
ACC and NMI of RAMSC with different selection of parameter λ. (A) NBA-NASCAR; (B) WebKB.

As for the parameter determination problem, we conduct experiments on two data sets, i.e., NBA-NASCAR and WebKB, for evaluation. Since we fix *k* = 5 and *p* = 1, only the balance parameter λ needs to be tuned when we use Eq (21) to construct **S**_1_. We vary it from {1, 2, 5, 10, 15, 20, 25, 30, 35, 40, 45, 50, 55, 60} and ACC and NMI are employed as the evaluation criterions. The results are shown in Fig 2.

When we use Eq (22) to construct **S**_2_, there is an additional parameter *γ*. To show the influence of λ and *γ* on RAMSC(*S*_2_), we vary λ from {2, 10, 20, 30, 40, 50, 60}, and *γ* is varied from {0.1, 0.3, 0.5, 0.7, 0.9, 1.1, 1.3, 1.5, 1.7, 1.9}. ACC is employed as the evaluation criterion.

**Fig 3:**
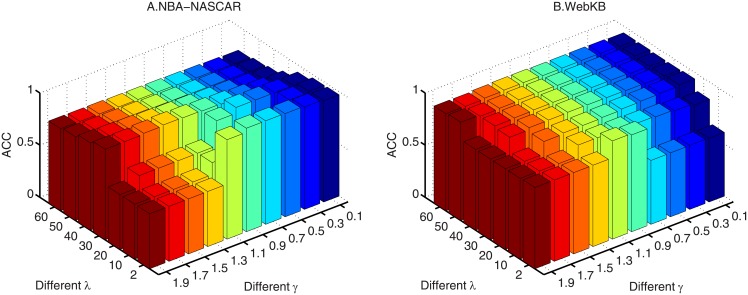
ACC and NMI of RAMSC(*S*_2_) with different combinations of parameters λ and *γ*. (A) NBA-NASCAR; (B) WebKB.

As we can see from the results in in Figs 2 and 3, it is clear that the final clustering results of RAMSC and RAMSC(*S*_2_) are affected by different λ and the combinations of λ and *γ*, respectively. Besides, on the two data sets, RAMSC has different optimal λ, and RAMSC(*S*_2_) has different optimal combination of λ and *γ* because the two data sets have different data characteristics.

## 6 Conclusion

In this paper, we have proposed a novel robust auto-weighted multi-view subspace clustering model, named RAMSC. This model can naturally assign suitable weights for each view and learn a common representation matrix. The common representation matrix can be used to construct a similarity matrix directly. Moreover, by imposing the structured sparsity norm, our method is robust to the inaccurate features. And the relative proof guarantees that the proposed method can converge to a local optimal solution. Experimental results on five data sets show that our proposed method enables a higher degree of accuracy than the state-of-the-art methods. However, there still remains several problems for future work:

- A series of relative methods need to be developed and systematically compared. The core idea of our method is to learn view weights automatically and find a high-quality common subspace representation matrix. Based on it, we list three possible ways to develop new relative methods. First, the smooth regularized terms of our method can be replaced by others; Second, the sparsity norm on error matrix can be considered to replace by other reasonable norms; Third, our method has no constraint, and some constraints on the common representation matrix or the error matrix can be added. According to specific applications, corresponding relative methods can be proposed.
- Another open problem lies in the selection of the parameters, especially in the balance parameter λ, which is still an unsolved problem in many learning algorithms. In this paper, we determine it empirically. Additional theoretical analysis is also needed for this topic.

## Supporting information

S1 Appendix RAMSC. A file contains matlab codes of RAMSC and the normalized datasets used in this paper.
(ZIP)
